# Myocardial protection in paediatric cardiac surgery: building an evidence-based strategy

**DOI:** 10.1308/rcsann.2023.0004

**Published:** 2023-05-30

**Authors:** NE Drury

**Affiliations:** ^1^ University of Birmingham, UK; ^2^ Birmingham Women’s and Children’s NHS Foundation Trust, UK

**Keywords:** Myocardial protection, Cardioplegia, Children, Cardiac surgery

## Abstract

Cardioplegia is fundamental to the surgical repair of congenital heart defects by protecting the heart against ischaemia/reperfusion injury, characterised by low cardiac output and troponin release in the early postoperative period. The immature myocardium exhibits structural, physiological and metabolic differences from the adult heart, with a greater sensitivity to calcium overload-mediated injury during reperfusion. Del Nido cardioplegia was designed specifically to protect the immature heart, is widely used in North America and may provide better myocardial protection in children; however, it has not been commercially available in the UK, where most centres use St Thomas’ blood cardioplegia. There are no phase 3 clinical trials in children to support one solution over another and this lack of evidence, combined with variations in practice, suggests the presence of clinical equipoise. The best cardioplegia solution for use in children, and the impact of age and other clinical factors remain unknown.

In this Hunterian lecture, I propose an evidence-based strategy to improve myocardial protection during cardiac surgery in children through: (1) conducting multicentre clinical trials of established techniques; (2) improving our knowledge of ischaemia/reperfusion injury in the setting of cardioplegic arrest; (3) applying this to drive innovation, moving beyond current cardioplegia solutions; (4) empowering personalised medicine, through combining clinical and genomic data, including ethnic diversity; and (5) understanding the impact of cardioplegic arrest on the late outcomes that matter to patients and their families.

## Introduction

During most surgery for congenital heart disease, it is necessary to stop the heart, allowing access to a still and bloodless field to enable repair of intracardiac defects while the patient is supported on cardiopulmonary bypass. A cross-clamp is placed across the proximal aorta, separating the coronary arteries from the rest of the systemic circulation, and cardioplegia solution administered, usually antegrade via the aortic root, leading to electromechanical quiescence.

Cardioplegia has been fundamental to arresting the heart and protecting against ischaemia/reperfusion injury during surgery for over 50 years, with approximately 2,500 cardiac surgical operations with cardioplegic arrest performed in children in the UK and Republic of Ireland each year.^1^ There is a perception that if the heart stops and restarts when needed, and the patient recovers to go home, the procedure has been a success and myocardial protection is a problem solved; yet myocardial injury occurs routinely following aortic cross-clamping in children, with the ubiquitous release of troponin in the early postoperative period,^2,3^ and this has been shown to strongly correlate with clinical outcomes including inotropic support, duration of ventilation, ventricular dysfunction and early death.^3^

Low cardiac output syndrome in the early postoperative period reflects the degree of myocardial injury or the presence of a major residual lesion, with a need for inotropic support to maintain adequate tissue perfusion. It is a major determinant of outcome after heart surgery in children, with around half of deaths in the early postoperative period attributed to either low cardiac output or failure to separate from bypass.^4^ Myocardial protection during surgery is therefore a key determinant of heart function and outcome.

Cardioplegic arrest is a profound moment in the life story of a heart, usually the first and only time that it will stop beating during a lifetime, and while this has become routine practice worldwide, its impact should not be ignored. There is a perception among many surgeons that it does not really matter which tonic is used as “they all stop the heart”^5^ but adequate protection is not the same as optimal protection. We do not understand enough about what happens during cardioplegic arrest, how well it really protects the heart from injury, which of the many available solutions offers the best protection for which patients or the long-term consequences of arresting the immature heart during multiple operations. In this Hunterian lecture, I describe an evidence-based approach to improving myocardial protection and outcomes in children undergoing cardiac surgery during the next decade.

## Protecting the immature myocardium

The immature myocardium has significant structural, physiological and metabolic differences from the adult heart, including sarcoplasmic reticulum development, mitochondrial density, substrate utilisation, calcium handling and antioxidant defences.^6^ It may be less tolerant of ischaemia and more sensitive to calcium overload-mediated injury during reperfusion, particularly in the presence of hypoxaemia.^2,7^ However, current paediatric cardioplegia techniques are derived primarily from adult or laboratory models and myocardial protection that is effective in adults may not be optimal for young children, especially neonates and those with chronic preoperative cyanosis.^8^

### del Nido cardioplegia

Originally patented in the early 1990s, del Nido cardioplegia is unique in that it was developed specifically to enhance protection of the paediatric myocardium.^9^ It is a modified depolarising solution, delivered with 20% by volume of fully oxygenated autologous blood from the bypass circuit. Like most other solutions, it causes arrest by elevating extracellular potassium but provides additional cellular protection through several other components, namely:

–  lidocaine, a sodium channel blocker that prevents intracellular sodium and calcium ion accumulation during arrest, and increases the refractory period of the cardiac myocyte;
–  mannitol, an oxygen-free radical scavenger that has osmotic effects to reduce myocardial oedema;
–  a lower proportion of autologous whole blood than other types of blood cardioplegia, which maintains physiological buffering via erythrocyte carbonic anhydrase but has only a trace calcium concentration, reducing myocardial accumulation during ischaemia.

There is extensive laboratory evidence to support the principle of del Nido cardioplegia for the immature myocardium. In a neonatal piglet model, Bolling *et al* demonstrated the superiority of a hypocalcaemic blood cardioplegia in hypoxic hearts, with better preservation of ventricular function and energetics.^7^ In rat hearts, van Emous *et al* showed that lidocaine reduced sodium influx during ischaemia, leading to improved functional and metabolic recovery.^10^ In large animal models, del Nido’s group demonstrated the benefits of this highly buffered, low-calcium, glycolysis-promoting solution over standard hyperkalaemic solutions in neonates, with improved myocardial contractility and oxidative metabolism.^11^ Del Nido cardioplegia is thereby customised to reduce the impact of ischaemia/reperfusion on the immature myocardium. Combined with the need for only a single dose in most cases, reducing interruptions to surgical flow has led to its increasing popularity in the US, with a recent survey finding it is preferred by 76% of centres performing complex surgery in neonates.^12^

### Lack of clinical trial evidence

**Figure 1:**
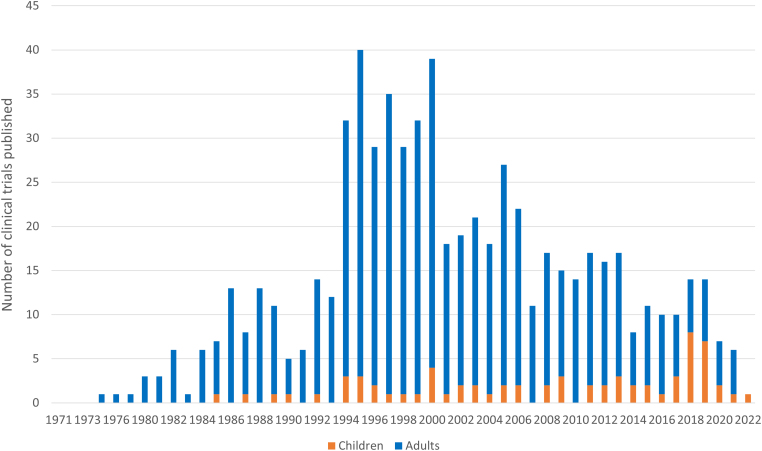
Clinical trials of cardioplegia published by year. PubMed^®^ search (performed on 19 April 2022): cardioplegia [tiab]; limit: clinical trial; results by year; paediatric studies identified using the improved CCG child filter

Cardioplegia used to be a hot topic for research in adult cardiac surgery. A simple PubMed^®^ search reveals that clinical trial publications on cardioplegia peaked in the mid-1990s but have since declined to only a handful each year worldwide; in contrast, there have been few trials in children (Figure 1).

In our systematic review of randomised controlled trials of cardioplegia in children, we identified 26 studies that were exclusively single-centre, phase 2 trials, recruiting few patients (median: 48, interquartile range: 30–99) and at risk of systematic bias.^13^ The most frequent comparison was blood versus crystalloid in ten trials, with only two comparing del Nido with St Thomas’ blood cardioplegia. The most common endpoints were biomarkers of myocardial injury (*n*=17, 65%), inotrope requirements (*n*=15, 58%) and length of stay in the intensive care unit (*n*=11, 42%). However, the heterogeneity of patients, interventions and reported outcome measures prohibited meta-analysis. Of concern, these trials included only 21 neonates (1.4%), a high-risk group in whom the effects of cardioplegia are less well understood.^6^ We concluded that the current literature contains no late-phase trials, and the small size, inconsistent use of endpoints and poor quality of reported trials provides a limited evidence base to guide patient care.

Several more recent trials have compared del Nido with other blood cardioplegia but all were small with significant methodological issues, including poor design, lack of sample size calculation and inadequate or no blinding. The best cardioplegia solution for children therefore remains unknown.

### Variations in clinical practice

Many types of cardioplegia solution are available worldwide and there is wide variation in their use. In a 2012 survey of Congenital Heart Surgeons’ Society members in North America,^14^ 86% of respondents preferred blood cardioplegia, with del Nido the most popular (38%); the next most frequent was “other” (34%), custom-mixed solutions unique to an individual centre, effectively a “none of the above” homebrew with little or no evidence to support its use.^13^ Most surgeons used their “standard” solution across all ages, suggesting that their strategy was determined by surgeon factors, not patient factors. Similarly, in the 2016 international survey of paediatric perfusion practice, there were wide variations in practice, with del Nido predominant in North America, depolarising solutions used widely elsewhere, and Custodiol^®^ HTK common across continental Europe and Central/South America.^15^ Myocardial protection strategy was therefore strongly influenced by geographical rather than patient factors.

In our recent survey of practice in the UK and Ireland, we found that St Thomas’ blood cardioplegia (Harefield preparation) was used routinely by 59% of surgeons from eight centres (67%), with another 22% using similar types of blood cardioplegia.^16^ No centre used del Nido cardioplegia as it has not been commercially available in the UK. However, 91% of surgeons would be willing to randomise patients to del Nido in a clinical trial, with the combination of del Nido and St Thomas’ blood cardioplegia having the greatest acceptability.

These variations in practice, driven by a lack of high-quality evidence to inform clinical decision making, suggest the presence of clinical equipoise. Which cardioplegia solution is best for children, how does this change with age and what is the impact of other clinical factors, such as prematurity or chronic cyanosis? This knowledge would enable the care of the child undergoing surgery to be individualised, could reduce perioperative myocardial injury, morbidity and costs, and may improve long-term cardiac function and quality of life. Indeed, the recent James Lind Alliance Priority Setting Partnership in Congenital Heart Disease identified improving organ protection during surgery as the number one priority for research in children.^17^

## Building an evidence-based strategy

In order to advance research in paediatric myocardial protection that can translate into improved patient outcomes, I propose a systematic approach to generate better evidence in several key areas, as outlined below.

### Multicentre clinical trials of established techniques

Despite being fundamental to most cardiac surgery, there are no published phase 3 clinical trials of cardioplegia in children.^13^ Indeed, most paediatric cardiac surgery trials are small, single-centre studies of low value and uncertain quality, providing a limited evidence base for contemporary practice.^18^

Surgical trials in children are challenging but can be achieved with thoughtful trial design, committed leadership, a collaborative approach and engagement with families to better understand their decision making.^19^ The single-ventricle reconstruction (SVR) trial randomised 555 neonates with single-ventricle lesions (84% of those eligible) undergoing the Norwood procedure to either a modified Blalock–Taussig shunt or a right ventricle-to-pulmonary artery shunt; the study was carried out across 15 sites over 3 years.^20^ This landmark trial demonstrated both the power of collaboration to increase sample size in a rare condition and the willingness of surgeons and parents to randomise children to different treatments, even in high-risk, complex neonatal cardiac surgery, when there is clinical equipoise.

The del Nido versus St Thomas’ blood cardioplegia in the young trial (DESTINY, ISRCTN 13638147) is a UK multicentre, individually randomised clinical trial that is funded by the British Heart Foundation and opened to recruitment in February 2022. It is evaluating whether del Nido cardioplegia improves myocardial protection, compared with St Thomas’ blood cardioplegia, in children undergoing surgery by reducing myocardial injury, shortening ischaemic time, improving clinical outcomes and modulating heart metabolism during ischaemia. Informed by our survey of practice, it is designed and conducted with extensive patient and public involvement, with broad inclusion and few exclusion criteria to maximise the applicability of its findings.

In developing the DESTINY trial, we partnered with Stockport Pharmaceuticals, a National Health Service (NHS) manufacturing pharmacy, to produce del Nido cardioplegia with an extended 12-month refrigerated shelf life (significantly beyond the 45 days limit for the US commercial product) so that the trial is evaluating a product that is sustainable in the NHS. The trial will also demonstrate the ability of the UK paediatric cardiac surgery community to conduct a complex, multicentre surgical trial and provide a platform for future trials.

### Ischaemia/reperfusion injury

In order to improve treatments, we need to better understand the pathophysiology of the disease. In their landmark paper, Chouchani *et al* showed that tissue ischaemia leads to local accumulation of succinate (a citric acid cycle metabolite) through reversal of succinate dehydrogenase, fumarate overflow from purine nucleotide breakdown and partial reversal of the malate–aspartate shuttle.^21^ On reperfusion, the accumulated succinate is rapidly reoxidised, driving a burst in production of reactive oxygen species by reverse electron transport at mitochondrial complex I, and leading to widespread oxidative damage, opening of the mitochondrial permeability transition pore and cell death.

However, the mechanism of ischaemia/reperfusion injury in cardioplegic arrest is different from that of classical models of global ischaemia; this is clear from the outcome, as an hour of the former is usually well tolerated while a similar period of the latter leads to widespread myocardial infarction. Our study of cardioplegia in a Langendorff mouse model, analysing the whole heart using ultra-performance liquid chromatography–mass spectrometry, demonstrates that ischaemic succinate accumulation does not occur during a moderate period of cardioplegic arrest and suggests a different mechanism for subsequent injury (unpublished data).

The classic studies by Buckberg *et al* demonstrated that electromechanical arrest reduces myocardial oxygen uptake and that hypothermia leads to a further stepwise reduction.^22^ The cooled, empty, arrested heart had an oxygen consumption of approximately 3.5% of the perfused beating heart but importantly, this was not zero, signalling an ongoing basal cellular energy requirement during arrest. Cardioplegia therefore modulates the impact of ischaemia/reperfusion, extending the period of ischaemic tolerance but with ongoing, time-dependent myocardial injury as longer aortic cross-clamp time is associated with worse outcomes.^2^ Consequently, we need to better understand the impact of cardioplegia on the mechanisms of ischaemia/reperfusion, how the associated injury could be further mitigated and the effect of clinical factors, such as cyanosis. Does the hypoxic stress of chronic cyanosis have a preconditioning effect on the heart prior to surgery^23^ or does it predispose the myocardium to start ischaemia in a worse metabolic state?^8^

### Innovation

The release of troponin following reperfusion reflects myocardial injury with all current protective strategies and so these techniques are not a silver bullet. We know that the immature myocardium is different from the adult heart, including substrate utilisation, calcium handling and antioxidants, but we have little insight into when the transition to the adult phenotype occurs, how this process is regulated or what factors may modulate it. Evolving our understanding of myocardial maturation and the interaction with ischaemia/reperfusion will allow us to move beyond current cardioplegia solutions, developing new and innovative strategies to maintain homeostasis, harness innate protective mechanisms and thereby reduce the impact of surgery.

One such approach that has gained considerable interest over the past 20 years is remote ischaemic preconditioning (RIPC), although its role as an adjunct to cardioplegia during surgery remains unclear. This simple, low-cost and readily available technique showed great promise in early preclinical studies but has failed to translate into clinical practice, with trials in both children and adults producing mixed results.^23–25^ While the precise mechanism of RIPC is not fully understood, there are several practical reasons why these trials may have failed, including subclinical reperfusion during ischaemia and the use of propofol anaesthesia, which interferes with the preconditioning pathway. Our two-centre bilateral remote ischaemic conditioning in children trial (BRICC, ISRCTN 12923441) is due to report shortly and will shed more light on the value of RIPC in young children, with or without preoperative cyanosis, undergoing surgery for congenital heart disease.^26^

### Personalised medicine

Many surgeons use their “standard” approach to cardioplegia in most if not all cases, determined by their personal preferences and local practice.^12,14–16^ Nevertheless, patients differ by age, presence and extent of cyanosis, and a multitude of genetic factors. For this reason, a one-size-fits-all approach is unlikely to be optimal. Cardiac surgery is already a personalised specialty, delivering a bespoke operation to match the patient’s unique anatomy and requirements, so why not optimise myocardial protection through a tailor-made strategy for each patient, considering their individual age, physiological and genetic characteristics? Until recently, this may have sounded like a step too far but in 2023, precision oncology has already become the standard of care for cancer treatment. Next-generation sequencing molecular analysis of tumours is being used to guide adjuvant therapy,^27^ through clinical trials and sharing of linked molecular/clinical datasets, and machine learning to refine patient selection and better predict outcome.

Genomic analysis in the clinic will grow in importance in cardiac surgery too. The development of an NHS genomic medicine service supported by supraregional genomic laboratory hubs and expansion of the national genomic test directory will rapidly enable whole genome sequencing to become embedded across routine clinical care. Identifying genomic factors that have an impact on myocardial protection, combined with clinical data linked to outcomes, will improve our understanding of genotype–phenotype interactions and selection of the optimal strategy for individual patients.

In the UK, the South Asian population is affected disproportionately by congenital heart disease and experiences worse outcomes from surgery than White ethnic groups, which is only partially explained by socioeconomic deprivation and access to healthcare.^28^ Such ethnic disparity is likely to have a basis in genomic diversity, with population demography, genetic drift and environmental adaptation over hundreds of generations translating into differing cellular responses to therapies.^29^ Given that European ancestries dominate genomic research (with South Asians making up <2% of participants in genome-wide association studies), it is essential that diverse and under-represented individuals are included in future genomic cohorts in order to better understand such variation and thereby improve outcomes.

### Impact on late outcomes

Beyond early postoperative survival, patients and families focus on long-term quality of life: how do they feel and what can they do? Nevertheless, there are no studies on the effects of myocardial protection strategies on daily symptoms, exercise capacity or late systolic and diastolic ventricular function. Are there differences between cardioplegia types? What is the impact of multiple operations during their life course? These outcomes are challenging to assess owing to the long interval between surgery and endpoints, with loss to follow-up, delayed return on funding and potentially comparing children of different ages. However, it should be an important consideration in large, well-funded trials, following the example of the Pediatric Heart Network’s SVR III extension trial, evaluating ventricular function in later school-age children (aged >10 years) following the Norwood operation as a neonate.^30^

## Conclusions

Cardioplegia is fundamental to the surgical management of children with congenital heart disease. Despite this, myocardial protection is not a problem that has been solved. Myocardial injury occurs routinely following every open heart operation with cardioplegic arrest, suggesting the need for renewed focus and innovation to improve patient outcomes. The immature myocardium is different from the adult phenotype and there is limited evidence to support one cardioplegia solution over another. While St Thomas’ blood cardioplegia is the most widely used in the UK, del Nido solution was specifically designed in the US to protect the paediatric heart and may provide better protection.

In this Hunterian lecture, I have proposed a systematic approach to improving myocardial protection in children, through multicentre clinical trials and better understanding of the pathophysiological process, in order to establish which of the current solutions is best for which patient, drive innovation and look beyond perioperative outcomes. An anatomically appropriate operation should be matched with physiologically optimised myocardial protection, and by embracing the era of personalised medicine we will continue to improve care and outcomes for our patients and their families.

## References

[1] National Institute for Cardiovascular Outcomes Research. *National Congenital Heart Disease Audit (NCHDA): 2021 Summary Report*. London: HQIP; 2021.

[2] Taggart DP, Hadjinikolas L, Hooper J *et al.* Effects of age and ischemic times on biochemical evidence of myocardial injury after pediatric cardiac operations. *J Thorac Cardiovasc Surg* 1997; **113**: 728–735. 9104982 10.1016/S0022-5223(97)70231-9

[3] Mildh LH, Pettilä V, Sairanen HI, Rautiainen PH. Cardiac troponin T levels for risk stratification in pediatric open heart surgery. *Ann Thorac Surg* 2006; **82**: 1643–1649. 17062219 10.1016/j.athoracsur.2006.05.014

[4] Gaies M, Pasquali SK, Donohue JE *et al.* Seminal postoperative complications and mode of death after pediatric cardiac surgical procedures. *Ann Thorac Surg* 2016; **102**: 628–635. 27154145 10.1016/j.athoracsur.2016.02.043 PMC4958574

[5] Chen JM. Who makes the best martini? *Semin Thorac Cardiovasc Surg* 2017; **29**: 375–376. 28917526 10.1053/j.semtcvs.2017.09.004

[6] Doenst T, Schlensak C, Beyersdorf F. Cardioplegia in pediatric cardiac surgery: do we believe in magic? *Ann Thorac Surg* 2003; **75**: 1668–1677. 12735611 10.1016/s0003-4975(02)04829-4

[7] Bolling K, Kronon M, Allen BS *et al.* Myocardial protection in normal and hypoxically stressed neonatal hearts: the superiority of hypocalcemic versus normocalcemic blood cardioplegia. *J Thorac Cardiovasc Surg* 1996; **112**: 1193–1200. 8911315 10.1016/S0022-5223(96)70132-0

[8] Imura H, Caputo M, Parry A *et al.* Age-dependent and hypoxia-related differences in myocardial protection during paediatric open heart surgery. *Circulation* 2001; **103**: 1551–1556. 11257084 10.1161/01.cir.103.11.1551

[9] Matte GS, del Nido PJ. History and use of del Nido cardioplegia solution at Boston Children’s Hospital. *J Extra Corpor Technol* 2012; **44**: 98–103. 23198389 PMC4557532

[10] van Emous JG, Nederhoff MG, Ruigrok TJ, van Echteld CJ. The role of the Na+ channel in the accumulation of intracellular Na+ during myocardial ischemia: consequences for post-ischemic recovery. *J Mol Cell Cardiol* 1997; **29**: 85–96. 9040024 10.1006/jmcc.1996.0254

[11] McGowan FX, Cao-Danh H, Takeuchi K *et al.* Prolonged neonatal myocardial preservation with a highly buffered low-calcium solution. *J Thorac Cardiovasc Surg* 1994; **108**: 772–779. 7934115

[12] Reagor JA, Clingan S, Kulat BT *et al.* The Norwood Stage I procedure – conduct of perfusion: 2017 survey results from NPC-QIC member institutions. *Perfusion* 2018; **33**: 667–678. 29963965 10.1177/0267659118781173

[13] Drury NE, Yim I, Patel AJ *et al.* Cardioplegia in paediatric cardiac surgery: a systematic review of randomized controlled trials. *Interact Cardiovasc Thorac Surg* 2019; **28**: 144–150. 29947787 10.1093/icvts/ivy199 PMC6328004

[14] Kotani Y, Tweddell J, Gruber P *et al.* Current cardioplegia practice in pediatric cardiac surgery: a North American multiinstitutional survey. *Ann Thorac Surg* 2013; **96**: 923–929. 23915588 10.1016/j.athoracsur.2013.05.052

[15] Walcƶak A, Klein T, Voss J *et al.* International pediatric perfusion practice: 2016 survey results. *J Extra Corpor Technol* 2021; **53**: 7–26. 33814602 10.1182/ject-2000033 PMC7995632

[16] Drury NE, Horsburgh A, Bi R *et al.* Cardioplegia practice in paediatric cardiac surgery: a UK & Ireland survey. *Perfusion* 2019; **34**: 125–129. 30095360 10.1177/0267659118794343 PMC6378396

[17] Drury NE, Herd CP, Biglino G *et al.* Research priorities in children and adults with congenital heart disease: a James Lind Alliance Priority Setting Partnership. *Open Heart* 2022; **9**: e002147. 36600635 10.1136/openhrt-2022-002147 PMC9843188

[18] Drury NE, Patel AJ, Oswald NK *et al.* Randomized controlled trials in children’s heart surgery in the 21st century: a systematic review. *Eur J Cardiothorac Surg* 2018; **53**: 724–731. 29186478 10.1093/ejcts/ezx388 PMC5848812

[19] Drury NE, Menzies JC, Taylor CJ *et al.* Understanding parents’ decision-making on participation in clinical trials in children’s heart surgery: a qualitative study. *BMJ Open* 2021; **11**: e044896. 10.1136/bmjopen-2020-044896 PMC7907877 33622954

[20] Ohye RG, Sleeper LA, Mahony L *et al.* Comparison of shunt type in the Norwood procedure for single-ventricle lesions. *N Engl J Med* 2010; **362**: 1980–1992. 20505177 10.1056/NEJMoa0912461 PMC2891109

[21] Chouchani ET, Pell VR, Gaude E *et al.* Ischaemic accumulation of succinate controls reperfusion injury through mitochondrial ROS. *Nature* 2014; **515**: 431–435. 25383517 10.1038/nature13909 PMC4255242

[22] Buckberg GD, Brazier JR, Nelson RL *et al.* Studies of the effects of hypothermia on regional myocardial blood flow and metabolism during cardiopulmonary bypass. I. The adequately perfused beating, fibrillating, and arrested heart. *J Thorac Cardiovasc Surg* 1977; **73**: 87–94. 831012

[23] McCrindle BW, Clarizia NA, Khaikin S *et al.* Remote ischemic preconditioning in children undergoing cardiac surgery with cardiopulmonary bypass: a single-center double-blinded randomized trial. *J Am Heart Assoc* 2014; **3**: e000964. 25074698 10.1161/JAHA.114.000964 PMC4310383

[24] Wu Q, Wang T, Chen S *et al.* Cardiac protective effects of remote ischaemic preconditioning in children undergoing tetralogy of Fallot repair surgery: a randomized controlled trial. *Eur Heart J* 2018; **39**: 1028–1037. 28329231 10.1093/eurheartj/ehx030 PMC6018784

[25] Hausenloy DJ, Candilio L, Evans R *et al.* Remote ischemic preconditioning and outcomes of cardiac surgery. *N Engl J Med* 2015; **373**: 1408–1417. 26436207 10.1056/NEJMoa1413534

[26] Drury NE, Bi R, Woolley RL *et al.* The Bilateral Remote Ischaemic Conditioning in Children (BRICC) trial: protocol for a two-centre, double-blind, randomised controlled trial in young children undergoing cardiac surgery. *BMJ Open* 2020; **10**: e042176. 10.1136/bmjopen-2020-042176 PMC7542918 33033035

[27] Cardoso F, van't Veer LJ, Bogaerts J *et al.* 70-Gene signature as an aid to treatment decisions in early-stage breast cancer. *N Engl J Med* 2016; **375**: 717–729. 27557300 10.1056/NEJMoa1602253

[28] Knowles RL, Ridout D, Crowe S *et al.* Ethnic-specific mortality of infants undergoing congenital heart surgery in England and Wales. *Arch Dis Child* 2019; **104**: 844–850. 30824491 10.1136/archdischild-2018-315505

[29] Gurdasani D, Barroso I, Zeggini E, Sandhu MS. Genomics of disease risk in globally diverse populations. *Nat Rev Genet* 2019; **20**: 520–535. 31235872 10.1038/s41576-019-0144-0

[30] Goldberg CS, Gaynor JW, Mahle WT *et al.* The pediatric heart network’s study on long-term outcomes of children with HLHS and the impact of Norwood Shunt type in the single ventricle reconstruction trial cohort (SVRIII): design and adaptations. *Am Heart J* 2022; **254**: 216–227. 36115392 10.1016/j.ahj.2022.09.005

